# Multivariate association between brain function and eating disorders using sparse canonical correlation analysis

**DOI:** 10.1371/journal.pone.0237511

**Published:** 2020-08-12

**Authors:** Hyebin Lee, Bo-yong Park, Kyoungseob Byeon, Ji Hye Won, Mansu Kim, Se-Hong Kim, Hyunjin Park

**Affiliations:** 1 Department of Electrical and Computer Engineering, Sungkyunkwan University, Suwon, Korea; 2 Center for Neuroscience Imaging Research, Institute for Basic Science (IBS), Suwon, Korea; 3 McConnell Brain Imaging Centre, Montreal Neurological Institute and Hospital, McGill University, Montreal, Quebec, Canada; 4 Department of Biostatistics, Epidemiology and Informatics, University of Pennsylvania, Philadelphia, Pennsylvania, United States of America; 5 Department of Family Medicine, St. Vincent's Hospital, College of Medicine, The Catholic University of Korea, Suwon, Korea; 6 School of Electronic and Electrical Engineering, Sungkyunkwan University, Suwon, Korea; Texas Tech University, UNITED STATES

## Abstract

Eating disorder is highly associated with obesity and it is related to brain dysfunction as well. Still, the functional substrates of the brain associated with behavioral traits of eating disorder are underexplored. Existing neuroimaging studies have explored the association between eating disorder and brain function without using all the information provided by the eating disorder related questionnaire but by adopting summary factors. Here, we aimed to investigate the multivariate association between brain function and eating disorder at fine-grained question-level information. Our study is a retrospective secondary analysis that re-analyzed resting-state functional magnetic resonance imaging of 284 participants from the enhanced Nathan Kline Institute-Rockland Sample database. Leveraging sparse canonical correlation analysis, we associated the functional connectivity of all brain regions and all questions in the eating disorder questionnaires. We found that executive- and inhibitory control-related frontoparietal networks showed positive associations with questions of restraint eating, while brain regions involved in the reward system showed negative associations. Notably, inhibitory control-related brain regions showed a positive association with the degree of obesity. Findings were well replicated in the independent validation dataset (n = 34). The results of this study might contribute to a better understanding of brain function with respect to eating disorder.

## Introduction

Eating disorder is one of the important factors underlying obesity [1–3], which is related to type 2 diabetes, cardiovascular diseases, stroke, and cancers [3–5]. Previous neuroimaging studies have shown that eating disorders were highly associated with the executive function of people with obesity [3,6–9]. Siep et al. found that an individual’s appetite was regulated by the inhibitory control centers of the brain [9]. Val-Laillet et al. reported a significant relationship between eating disorder and altered cognitive- and reward-related brain systems [3]. Our previous studies also demonstrated that executive- and inhibitory-related brain regions had strong associations with eating disorders in people with obesity [6–8]. These studies collectively suggested that neurologic dysfunctions were highly related to the eating disorders of people with obesity.

The association between brain alterations and eating disorders can be effectively explored using neuroimaging modalities such as functional magnetic resonance imaging (fMRI), positron emission tomography (PET), and single-photon emission computed tomography (SPECT) [3,9–11]. However, most existing neuroimaging studies did not make full use of the available information regarding participants’ eating disorders [6–8]. They used summary scores instead of the responses to the full list of questions. For example, the Three-Factor Eating Questionnaire (TFEQ) quantifies behavioral traits of eating disorders and has a total of 51 questions condensed into three factors of dietary restraint, disinhibition, and hunger scores [12,13]. The Eating Disorder Examination Questionnaire (EDE-Q) evaluates the psychopathology of eating disorders and has a total of 34 questions condensed into four factors of dietary restraint, eating concern, shape concern, and weight concern [14,15]. This approach allows researchers to quantify an individual’s eating disorders succinctly, but it does not utilize all the available information, and thus could lead to suboptimal findings regarding neurological alterations in the brain. Thus, considering all the questions in the questionnaire might allow us to better explore the associations between brain function and eating disorder.

Machine learning is widely used in neuroimaging and medical field, and we applied one of machine learning technique, canonical correlation analysis (CCA), for data analysis [16,17]. CCA is a useful method to identify the multivariate association between two types of high-dimensional features (e.g., neuroimaging and questionnaire) [18]. Sparse CCA (SCCA) is an extension of CCA, which aims to identify a sparse set of loading vectors in two types of features using a regularization technique [19–30]. SCCA could be more suitable than the regular CCA since it helps to reduce the overfitting problem, which is becoming more problematic with increased dimensionality of modern neuroimaging data. Associations between brain regions over the whole brain and all the questions of the eating disorder questionnaire could be effectively assessed using the SCCA approach. Especially, incorporating the information on eating disorders in a question-level might allow us to better model the complex associations between brain and behavioral traits of eating.

We adopted a functional connectivity analysis based on graph theory to estimate brain function [31,32]. Graph theory-based connectivity analysis is a representative method to identify the connection strengths of different brain regions, which requires graph nodes and edges [31,32]. Graph nodes are the brain regions defined by pre-defined structural or functional atlases and graph edges are the connections between two different nodes [31,32]. Graph theory-based functional connectivity analysis has been useful for explaining brain alterations in people with obesity, as well as identifying associations between the brain and eating disorders [6–8].

In the current study, we aimed to investigate the multivariate association between the functional connectivity of all brain regions and eating disorders at question-level using the SCCA approach. Our study is not limited to assess obesity-related eating disorders in the obese group, rather we aimed to associate functional connectivity of brain regions and behavioral patterns of eating disorders in a general population. The results of this study might contribute to a better understanding of brain function with respect to eating disorder.

## Materials and methods

### Imaging data and participants

This study was a retrospective analysis of anonymized data and institutional review board (IRB) approval was obtained at Sungkyunwkan University. All data were obtained with informed written consent in accordance with established human subject research procedures expressed in the Declaration of Helsinki. Our study was performed in full accordance with the local IRB guidelines. For the discovery set, the T1-weighted structural MRI and resting-state fMRI (rs-fMRI) data were obtained from the enhanced Nathan Kline Institute-Rockland Sample database [33]. All imaging data were obtained using a 3-T Siemens Magnetom Trio Tim scanner. The scanning parameters of the T1-weighted structural data were as follows: repetition time (TR) = 1900 ms, echo time (TE) = 2.52 ms, flip angle = 9°, field-of-view (FOV) = 250 mm × 250 mm, 1 mm^3^ voxel resolution, and 176 slices. Those of the rs-fMRI data were as follows: TR = 645 ms, TE = 30 ms, flip angle = 60°, FOV = 222 mm × 222 mm, 3 mm^3^ voxel resolution, 40 slices, and 900 volumes. Among the total of 650 participants, 320 participants have full demographic information, obesity-related clinical scores (body mass index [BMI] and waist-to-hip ratio [WHR]), and TFEQ scores. BMI and WHR are calculated from the vitals (i.e., height, waist measurement, hip measurement, and weight) that were recorded by staff with a standardized procedure. Thirty-six subjects with medical conditions (e.g., attention deficit hyperactivity disorder, balance dysfunction with severe motion sensitivity, depression, diabetes, high blood pressure, high cholesterol, and hypoglycemia) were excluded and total 284 participants were considered in this study. Detailed demographic information is reported in Table 1. The study participants had a large variation in BMI as stated in Table 1 and the S1 Fig in S1 File.

**Table 1:** Demographic information of study participants.

Dataset		eNKI dataset (n = 284)	Vincent dataset (n = 34)
Information		Mean (standard deviation)	Mean (standard deviation)
**Age**		36.79 (13.63)	39.06 (10.76)
**Sex (male: female)**		106:178	17: 17
**BMI (kg/m**^**2**^**)**		27.30 (5.79)	29.56 (6.43)
**WHR**		0.83 (0.09)	0.92 (0.06)
**healthy weight: overweight: obesity**		119: 86: 79	3: 19: 12
**TFEQ/EAT-26 scores**	**Dietary restraint/Diet**	8.11 (4.96)	6.06 (4.82)
	**Disinhibition/Bulimia**	5.04 (3.36)	0.24 (0.50)
	**Hunger/Oral control**	4.48 (3.33)	1.76 (2.22)

Healthy weight group was defined as BMI value less than 25 kg/m^2^, overweight group was defined as BMI value between 25 and 30 kg/m^2^, and obesity group was defined as BMI value greater than or equal to 30 kg/m^2^. TFEQ has dietary restraint, disinhibition, and hunger scores and EAT-26 has diet, bulimia and food preoccupation, and oral control scores.

BMI, Body mass index; WHR, waist-to-hip ratio; TFEQ, Three-Factor Eating Questionnaire; EAT-26, Eating Attitudes Test.

Data obtained from Saint Vincent’s hospital in South Korea was used for validation. All data were obtained with informed written consent in accordance with established human subject research procedures expressed in the Declaration of Helsinki. Our study was performed in full accordance with the local IRB guidelines. The dataset includes T1-weighted data, rs-fMRI, full demographic information, BMI, WHR, and Eating Attitudes Test (EAT-26) scores of 34 participants [34]. The T1-weighted structural MRI and rs-fMRI data were scanned using a Siemens Magnetom 3T scanner housed at St. Vincent’s Hospital. The image acquisition parameters of the T1-weighted structural MRI were as follows: TR = 1900 ms; TE = 2.46 ms; flip angle = 9°; FOV = 250 mm × 250 mm; 1 mm^3^ voxel resolution; and 160 slices. Those of the rs-fMRI data were as follows: TR = 2490 ms; TE = 30 ms; flip angle = 90°; FOV = 220 mm × 220 mm; 3.4 × 3.4 × 3.0 mm^3^ voxel resolution; 36 slices; 150 volumes.

### Eating disorder scores

The eating disorder scores were measured using the TFEQ, which contains a total of 51 questions [12]. All the questions were assigned to one of the three factors of dietary restraint, disinhibition, or hunger. The question list of the TFEQ is stated in the Supplementary Tables in S1 File. Each question had a binary response and the score of each factor was calculated by summing the responses of the assigned questions. In this study, the response to each question was considered rather than the score for each factor.

EAT-26 is a reliable screening tool for eating disorders [34–38] and it was used to access the degree of eating disorders in the validation set. This has 26 questions and the answers are scored among 0, 1, 2, 3, 4, or 5 (correspond to never, rarely, sometimes, often, usually, or always respectively). Those scores are rescored into 0 to 3 and summed up into three subscales (diet, bulimia and food preoccupation, and oral control).

### Data preprocessing

**Fig 1:**
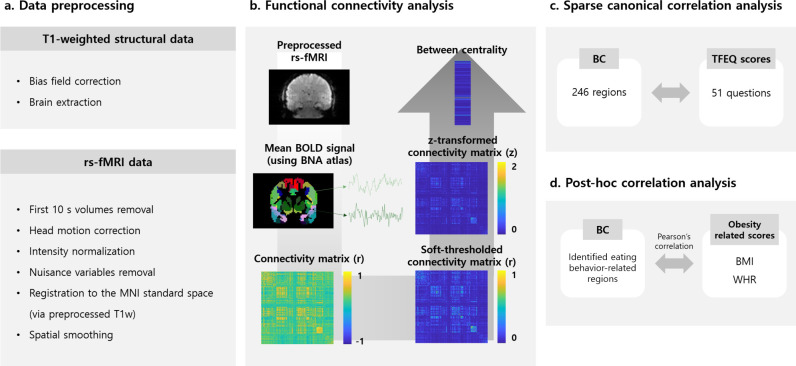
Overall flowchart of procedures in the study for the discovery set. (a) Data preprocessing pipeline of T1-weighted structural data and rs-fMRI data. (b) Functional connectivity analysis steps applied to preprocessed rs-fMRI data. The mean BOLD signal of each region as defined by the BNA atlas was calculated and the connectivity matrix was computed using Pearson’s correlation coefficients from the mean BOLD signal of two different regions. This connectivity matrix was soft-thresholded and then z-transformed. The between centrality (BC) values were estimated from this matrix. (c) Sparse canonical correlation analysis (SCCA) was adopted to find an association between brain function and eating disorders. The BC values of all 246 brain regions and 51 responses of the TFEQ assessment were considered. (d) The post-hoc correlation analysis was conducted between obesity-related scores and brain regions showing associations with eating disorders as result of (c). Pearson’s correlation was computed from the BC values of the corresponding brain regions and BMI/WHR scores.

The T1-weighted structural data and rs-fMRI data were preprocessed using a Fusion of Neuroimaging Preprocessing (FuNP) pipeline that integrates AFNI, FSL, and ANTs software [39–42]. The T1-weighted structural data were preprocessed as follows: the distortion arising from the magnetic field inhomogeneity was corrected and non-brain tissues were removed. The rs-fMRI data were preprocessed as follows: the volumes from the first 10 s of the scan were removed and distortions caused by head motions were corrected. Intensity normalization of the 4D volumes was applied and nuisance variables of the cerebrospinal fluid, white matter, head motion, and cardiac- and large-vein-related artifacts were removed using FIX software [43]. The artifact-removed rs-fMRI data were registered onto preprocessed T1-weighted structural data and then subsequently registered onto 3 mm isotropic Montreal Neurological Institute (MNI) standard space. Spatial smoothing with a full width at half maximum of 5 mm was applied. The preprocessing pipeline is summarized in Fig 1A.

### Functional connectivity analysis

Graph theory-based functional connectivity analysis was adopted in this study. Graph nodes were the brain regions defined by the Brainnetome atlas [44] and the graph edges were defined by Pearson’s correlation coefficients of time series between two different nodes. Recent resting-state fMRI studies suggested that inter-regional functional connectivity shows a small-world property linked with scale-free topology [45–47]. The soft-thresholding approach was applied to the correlation coefficients to satisfy the scale-free topology using the following formula: {(r+1)/2}^β^, where r is the correlation coefficient and β is the scale-free index that was set to six [48,49]. The soft-thresholded correlation coefficients were transformed to z-values using Fisher’s r-to-z transformation. The betweenness centrality, a graph measure estimating the importance of a given node, was calculated by counting the number of shortest paths between any two nodes that run through that node [31,32]. The steps for functional connectivity analysis are represented in Fig 1B.

### Association between functional connectivity of the brain and eating disorders

The multivariate association between the functional connectivity of the whole brain and eating disorders was explored using the SCCA approach. The betweenness centrality values of 246 brain regions and responses of 51 questions in TFEQ assessment were considered (Fig 1C). The goal of SCCA is to compute loading vectors (**u** and **v**) of two feature matrices, **X** and **Y**, by maximizing the correlation between the linear combinations of the features from two matrices **(Xu** and **Yv)**, where **X** is the betweenness centrality values of all brain regions (n × 246 regions), **Y** is the responses to all questions in the TFEQ assessment (n × 51 questions), and n is the number of participants. L1 regularization was used to control the sparse relationship between the two different feature matrices [50]. The objective function of SCCA can be defined as

$$\underset{u,v}{\max}\;u^{T}X^{T}Yv\;s.t.\;u^{T}X^{T}Xu\leq 1,v^{T}Y^{T}Yv\leq 1,{\left\|u\right\|}_{1}\leq c_{1},{\left\|v\right\|}_{1}\leq c_{2}.$$ (1)

We can rewrite the objective function as

$$\underset{u,v}{\min}\;-u^{T}X^{T}Yv+\beta_{u}{\left\|u\right\|}_{1}+\beta_{v}{\left\|v\right\|}_{1}\;s.t.\;u^{T}X^{T}Xu\leq 1,v^{T}Y^{T}Yv\leq 1.$$ (2)

Solving Eq (1) under the sparsity constraints lead to solving Eq (2). We initially tuned the regularization parameters (i.e., β_u_ and β_v_) using five-fold cross-validation where the built SCCA model from the training fold was tested using various beta values in the left-out fold to maximize the canonical correlation. The final beta values were determined by averaging them across the cross-validation. The SCCA is a multivariate approach, which aims to optimize the association between one set of variables (i.e., functional connectivity of various brain regions) and another set of variables (i.e., questions in the TFEQ) by maximizing the canonical correlation between the linear combination of a subset of each variable and relevant loading vector. This approach does not involve multiple tests of univariate statistics and therefore, we evaluated the model just once. Thus, there are no corrected p-values in that sense. Bootstrapping was performed in order to assess the reliability of the results, randomly selecting 80% of subjects for 1000 times. The loading vectors were computed for each bootstrapped subset and the frequently selected features were compared with those obtained from the full data.

### Correlation with obesity-related clinical scores

Post-hoc correlation analysis between the betweenness centrality values of the identified brain regions, which showed an association with eating disorders, and the obesity-related clinical scores of BMI and WHR was conducted at two levels, one at the large-scale network and the other at the smaller-scale region (Fig 1D). This was to explore whether brain regions were associated with obesity as well as eating disorders. The r- and p-values were calculated and the regions with significant correlation with BMI/WHR were selected (false discover rate [FDR] corrected p < 0.05).

### Validation of the association between brain regions and eating disorders

Independent validation was conducted using data of the Saint Vincent’s hospital in Suwon, South Korea. The betweenness centrality was calculated from rs-fMRI data and the same atlas was used. SCCA was applied to the centrality values of 246 brain regions and responses to 26 questions of EAT-26. The optimal regularization parameter was obtained in the same way described before, but three-fold cross-validation was used in parameter tuning due to the small sample size.

## Results

### Association between functional connectivity of the brain and eating disorders

**Fig 2:**
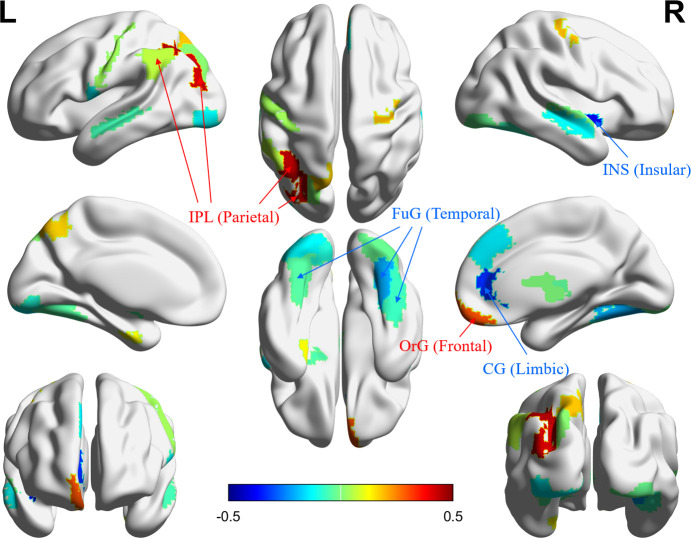
The coefficients of the loading vector for the brain visualized in atlas space. The color scale indicates a strong association with blue and red colors. The regions in red (strong positive correlation with dietary restraint) are mostly cognition-related regions. The regions in blue (strong negative correlation with dietary restraint) are mostly reward-related regions. IPL, inferior parietal lobule; OrG, orbital gyrus; INS, insula; FuG, fusiform gyrus; CG, cingulate gyrus.

Twenty-seven brain regions and 19 questions were selected as a result of the SCCA. The canonical correlation coefficient between two linear combinations of two features was 0.4945. The brain regions with a large magnitude of the loading vector (i.e., the strong association with TFEQ questions) were primarily located at the frontoparietal and limbic networks that process cognition- and reward-related functions (Fig 2). The questions in the TFEQ with a large loading vector magnitude were primarily involved in factor 1, dietary restraint, which indicates the ability to control food intake. Detailed information for selected features is stated in the Supplementary Tables in S1 File. Positive associations were observed between the questions of dietary restraint factor and cognition-related brain regions, while negative associations were found between the same factor with reward-related brain regions. The results indicate that the inhibitory function in cognition-related brain regions might regulate eating disorders, while hunger-related responses in reward-related brain regions could lead to a failure in dietary regulation.

Bootstrapping was performed for 1000 times and the frequently selected features were compared with those from the main results. Twenty-seven selected brain regions of the main results were compared with the top 27 frequently selected brain regions from the bootstrapping. A total of 21 regions (77.78%) overlapped. Nineteen selected questions of the main results were compared with the top 19 frequently selected questions from the bootstrapping. A total of 18 questions (94.74%) overlapped and overlapped features were mostly assigned in dietary restraint. The list of the most frequently selected features is stated in the Supplementary Tables in S1 File.

### Correlation with obesity-related clinical scores

To explore whether the eating disorder-related brain regions were associated with obesity, post-hoc correlation analysis between the betweenness centrality values of the networks in the behavioral domain of the identified regions and BMI/WHR was conducted. Twenty-seven identified brain regions were mapped into five behavioral domains which appeared dominantly in the identified regions (cognition-, reward-, sensorimotor-, visual-, and language-related region) and the averaged centrality values of each domain were computed. The results are shown in Table 2. The cognition-related network showed a significant correlation with WHR with r-value of 0.1588 and FDR corrected p-value of 0.0366. We further explored the association in six regions in the cognitive domain with WHR to give a fine-grained explanation of the results. Table 3 shows the results of the second correlation analysis. Two regions (rostal area 35/36 and caudal area 40) showed a significant correlation with WHR, indicating a significant association among cognition-related brain networks, eating disorders, and obesity.

**Table 2:** The correlation between the behavioral domain of the identified brain regions and obesity-related clinical scores.

Behavioral domain	Clinical score			
	BMI		WHR	
	r-value	p-value	r-value	p-value
***Cognition***	0.0969	0.2576	***0*.*1588***	*0*.0366 *
**Reward**	-0.0341	0.7091	-0.0480	0.6636
**Sensorimotor**	-0.0136	0.8191	0.0259	0.6636
**Visual**	-0.1185	0.2301	-0.1028	0.2095
**Language**	-0.0614	0.5040	-0.0357	0.6636

Significant results are reported in bold italic. BMI, body mass index; WHR, waist-to-hip ratio; p-values were corrected using FDR approach.

**Table 3:** The correlation between the identified cognition-related brain regions and WHR.

Atlas index	Region	Gyrus	Hemi-sphere	r-value	p-value
***109***	***rostral area 35/36***	***Parahippocampal Gyrus***	***L***	***0*.*2377***	*0*.0003 *
**127**	caudal area 7	Superior Parietal Lobule	L	-0.0197	0.7408
**135**	caudal area 39(PGp)	Inferior Parietal Lobule	L	0.1211	0.0828
**137**	rostrodorsal area 39(Hip3)	Inferior Parietal Lobule	L	0.0947	0.1670
***141***	***caudal area 40(PFm)***	***Inferior Parietal Lobule***	***L***	***0*.*1693***	*0*.0127 *
**209**	lateral superior occipital gyrus	lateral Occipital Cortex	L	0.0826	0.1983

Significant results are reported in bold italic. p-values were corrected using FDR approach.

### Validation of the association between brain regions and eating disorders

As a result of SCCA, 16 brain regions and eight questions were selected. The canonical correlation coefficient between two canonical features was 0.8851. The cognition- and reward-related regions showed a large magnitude of loading vectors and the ‘diet’ subscale had the largest magnitude in loading vectors. Thus, high associations between cognition-/reward-related brain regions and diet were observed in the validation. The diet subscale in EAT-26 is not equal to the dietary restraint in TFEQ at the subscale-level but it includes some questions related to dietary restraint and might be partially comparable. In this sense, these results of the validation were largely compatible with those of the discovery set. The list of selected features is stated in the Supplementary Tables in S1 File.

## Discussion

Despite extensive research [3,6–10], association between brain connectivity topology and behavioral patterns of eating disorder is underexplored, which makes it difficult to understand the underlying mechanisms of brain function related to eating disorders. Leveraging a multivariate association analysis, we established that reward and frontoparietal networks showed significant associations with eating disorders of dietary restraint. Regions involved in frontoparietal network positively related to restraint eating, while reward regions showed a negative association. These results support that increased inhibitory control of the brain may suppress errant eating behavior, while increased reward signaling induces loss of control for eating. Further association with the degree of obesity revealed a potential link between inhibitory control and obesity. Together, our work provides a new perspective on understanding functional connectivity topology associated with eating disorders.

In this current study, we adopted SCCA to identify highly contributable brain regions associated with eating disorders across a wide range of BMI. We found frontoparietal network to be related to restraint eating, supporting the role of this brain region for regulating inhibitory behavior [51–54]. In addition, reward-related brain regions showed significant associations with eating disorders as well. Unlike inhibition control-related brain regions, these regions are involved in hunger domain, which encodes food-related responses leading to an individual to feel hunger [55–60]. Previous neuroimaging studies proved that dysfunction in frontoparietal network broke the executive and inhibitory control system, yielding aberrant eating behaviors and subsequently weight gain [6,7,53,61–65]. Molecular studies support our findings in a biological context by suggesting atypical signaling from neuroendocrine factors of insulin, leptin, and ghrelin and corresponding gene expressions modulate perturbed inhibitory and reward systems, encouraging eating disorders [66,67]. These studies collectively suggest neural substrates of executive-control and reward-related brain regions that influence the behavioral traits of eating.

Our work adopted eating disorder scores at the question-level rather than the factor-wise scores. We hypothesized that using the full information provided in the question-wise responses (51 features) would be better suited for exploring the link between the brain and eating behaviors than using factor-wise scores (three features) due to the increased information content. Indeed, our previous study investigated the association between functional brain networks and eating behaviors in people with obesity using factor-wise scores and found the association with an effect size (i.e., Pearson’s r) of 0.2422 [6], which was largely improved in our current study (Pearson’s r = 0.4945). These results indicate that our approach could better select the combination of functional brain connectivity to be maximally linked to behavioral traits of eating disorders. Thus, the regions identified in our study might have higher levels of statistical confidence, although the identified regions were similar to those identified in previous studies. Interestingly, we found that the questions related to a similar behavioral trait, especially dietary restraint, showed clustered association with the brain, confirming previous studies that linked cognition- and reward-related brain networks to eating behaviors [3,6–9]. Together, our work supports that the TFEQ at question-level could be a reliable measurement for assessing eating disorders in conjunction with brain function.

Although we found significant associations between the identified brain regions and dietary restraint scores, no significant association was detected for disinhibition or hunger factors. This should be investigated further, but this inability might be due to the demographic characteristics of enrolled participants, in which obesity-related diseases were excluded. Further validations are required by recruiting participants with aberrant eating disorders.

Our study has several limitations. First, we only used betweenness centrality for quantifying complex brain networks. Other graph-related measures such as degree- and eigenvector-centrality, and efficiency might be used to associate the brain and eating behaviors. This is left to future work. Second, obesity is a multifactorial disorder affected by a wide range of factors, including eating behaviors, genetic factors, and other environmental factors [68,69]. Strict control of these factors was difficult since we obtained our data from an open database. Third, a longitudinal study that follows the changes in eating behaviors is needed to assess the stability of our findings. Fourth, patient demographic and eating disorder questionnaires were not exactly matched between discovery and validation sets (p-value < 0.05). Despite these potential confounds, we found largely consistent results between discovery and validations sets. However, a controlled validation set is needed to fully test the generalizability of our approach.

Here, we introduced a framework for associating functional brain connectivity and eating disorders at question-level. Harnessing a multivariate association analysis, SCCA, we identified brain regions in executive control and reward system are functionally associated with restraint eating in people with a wide range of BMI. Association between the functional connectivity and WHR suggested the potential role of functional variations in inhibitory control associated with obesity. Our work provides a new perspective on understanding the relationship between eating disorder and brain function.

## Supporting information

S1 File The list of questions from the TFEQ and the results of SCCA. The distribution of obesity-related clinical scores in the discovery set. The list of 51 questions of the TFEQ. The list of selected features as a result of SCCA for the discovery and validation set. The most frequently selected features from bootstrap.
(DOCX)
