# Supplementary material for: Multivariate association between brain function and eating disorders using sparse canonical correlation analysis
Source: PLoS One. 2020 Aug 12;15(8):e0237511. doi: 10.1371/journal.pone.0237511 (PMC7423138; doi:10.1371/journal.pone.0237511)
Supplement: S1 File — The distribution of obesity-related clinical scores in the discovery set. The list of 51 questions of the TFEQ. The list of selected features as a result of SCCA for the discovery and validation set. The most frequently selected features from bootstrap. (DOCX) [file pone.0237511.s001.docx]

**Supplementary Information**

**Title: Multivariate association between brain function and eating disorders using sparse canonical correlation analysis**

Authors: Hyebin Lee^1,2¶^, Bo-yong Park^3¶^, Kyoungseob Byeon^1,2^, Ji Hye Won^1,2^, Mansu Kim^4^, Se-Hong Kim^5^, Hyunjin Park^2,6*^

^1^ Department of Electrical and Computer Engineering, Sungkyunkwan University, Suwon, Korea

^2^ Center for Neuroscience Imaging Research, Institute for Basic Science (IBS), Suwon, Korea

^3^ McConnell Brain Imaging Centre, Montreal Neurological Institute and Hospital, McGill University, Montreal, Quebec, Canada

^4^ Department of Biostatistics, Epidemiology and Informatics, University of Pennsylvania, Philadelphia, Pennsylvania, United States of America

^5^ Department of Family Medicine, St. Vincent's Hospital, College of Medicine, The Catholic University of Korea, Suwon, Korea

^6^ School of Electronic and Electrical Engineering, Sungkyunkwan University, Suwon, 16419, Korea

*Corresponding author

E-mail: hyunjinp@skku.edu (HP)

^¶^These authors contributed equally to this work.

**S1 Figure. The distribution of body mass index (BMI) and waist-hip ratio (WHR) values for all participants in the discovery set.** (a) The distribution of BMI (b) The distribution of WHR


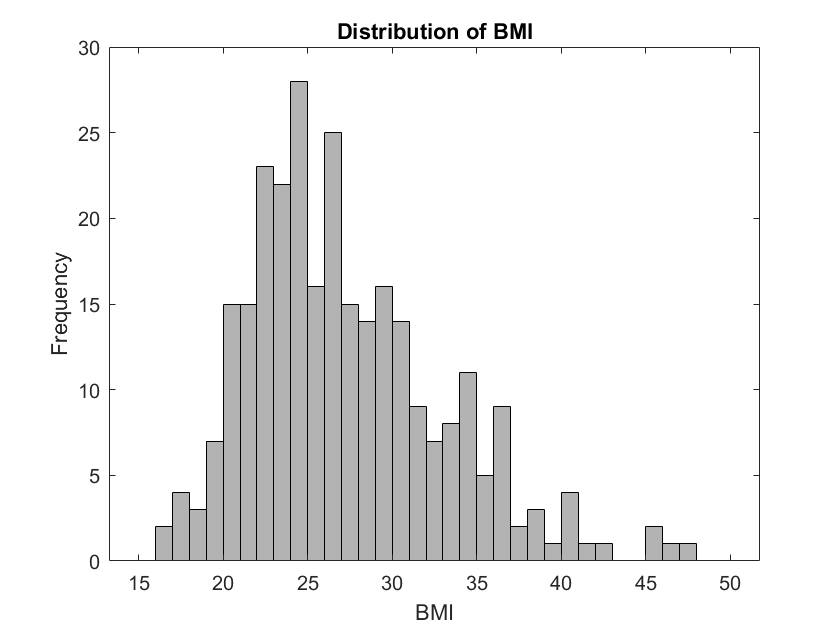


(a)


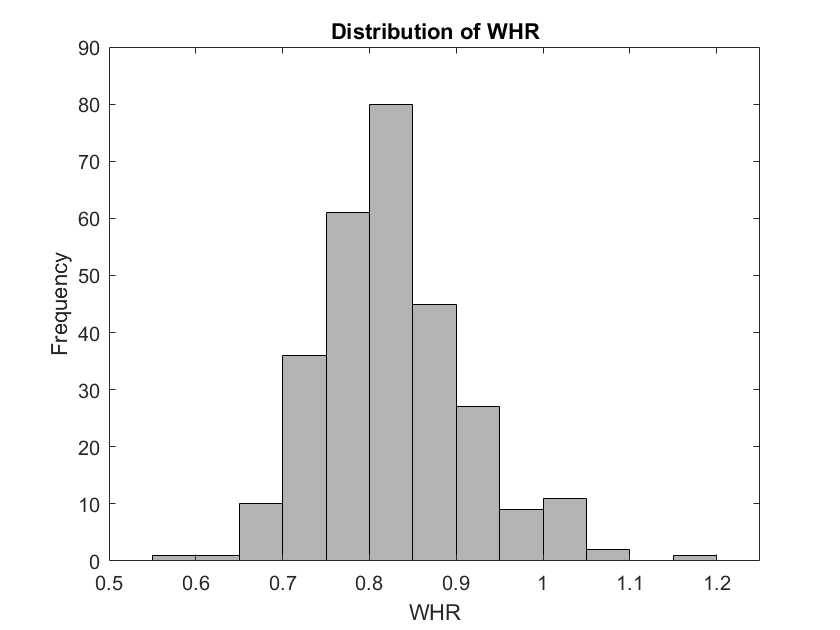


(b)

**Supplementary Table 1. List of questions from the TFEQ and corresponding factor.** Factor 1 is dietary restraint, factor 2 is disinhibition, and factor 3 is hunger.

| Index | Question | Factor |
| --- | --- | --- |
| 1 | When I smell a sizzling steak or see a juicy piece of meat, I find it very difficult to keep from eating, even if I have just finished a meal. | 2 |
| 2 | I usually eat too much at social occasions, like parties and picnics. | 2 |
| 3 | I am usually so hungry that I eat more than three times a day. | 3 |
| 4 | When I have eaten my quota of calories, I am usually good about not eating anymore. | 1 |
| 5 | Dieting is so hard for me because I just get too hungry. | 3 |
| 6 | I deliberately take small helpings as a means of controlling my weight. | 1 |
| 7 | Sometimes things just taste so good that I keep on eating even when I am no longer hungry. | 2 |
| 8 | Since I am often hungry, I sometimes wish that while I am eating, an expert would tell me that I have had enough or that I can have something more to eat. | 3 |
| 9 | When I feel anxious, I find myself eating. | 2 |
| 10 | Life is too short to worry about dieting. | 1 |
| 11 | Since my weight goes up and down, I have gone on reducing diets more than once. | 2 |
| 12 | I often feel so hungry that I just have to eat something. | 3 |
| 13 | When I am with someone who is overeating, I usually overeat too. | 2 |
| 14 | I have a pretty good idea of the number of calories in common food. | 1 |
| 15 | Sometimes when I start eating, I just can't seem to stop. | 2 |
| 16 | It is not difficult for me to leave something on my plate. | 2 |
| 17 | At certain times of the day, I get hungry because I have gotten used to eating then. | 3 |
| 18 | While on a diet, if I eat food that is not allowed, I consciously eat less for a period of time to make up for it. | 1 |
| 19 | Being with someone who is eating often makes me hungry enough to eat also. | 3 |
| 20 | When I feel blue, I often overeat. | 2 |
| 21 | I enjoy eating too much to spoil it by counting calories or watching my weight. | 1 |
| 22 | When I see a real delicacy, I often get so hungry that I have to eat right away. | 3 |
| 23 | I often stop eating when I am not really full as a conscious means of limiting the amount that I eat. | 1 |
| 24 | I get so hungry that my stomach often seems like a bottomless pit. | 3 |
| 25 | My weight has hardly changed at all in the last ten years. | 2 |
| 26 | I am always hungry so it is hard for me to stop eating before I finish the food on my plate. | 3 |
| 27 | When I feel lonely, I console myself by eating. | 2 |
| 28 | I consciously hold back at meals in order not to gain weight | 1 |
| 29 | I sometimes get very hungry late in the evening or at night. | 3 |
| 30 | I eat anything I want, any time I want. | 1 |
| 31 | Without even thinking about it, I take a long time to eat. | 2 |
| 32 | I count calories as a conscious means of controlling my weight. | 1 |
| 33 | I do not eat some foods because they make me fat. | 1 |
| 34 | I am always hungry enough to eat at any time. | 3 |
| 35 | I pay a great deal of attention to changes in my figure. | 1 |
| 36 | While on a diet, if I eat a food that is not allowed, I often then splurge and eat other high-calorie foods. | 2 |
| 37 | How often are you dieting in a conscious effort to control your weight. | 1 |
| 38 | Would a weight fluctuation of 5 lbs affect the way you live your life? | 1 |
| 39 | How often do you feel hungry? | 3 |
| 40 | Do your feelings of guilt about overeating help you to control your food intake? | 1 |
| 41 | How difficult would it be for you to stop eating halfway through dinner and not eat for the next few hours? | 3 |
| 42 | How conscious are you of what you are eating? | 1 |
| 43 | How frequently do you avoid 'stocking up' on tempting foods? | 1 |
| 44 | How likely are you to shop for low calorie foods? | 1 |
| 45 | Do you eat sensibly in front of others and splurge alone? | 2 |
| 46 | How likely are you to consciously eat slowly in order to cut down on how much you eat? | 1 |
| 47 | How frequently do you skip dessert because you are no longer hungry? | 3 |
| 48 | How likely are you to consciously eat less than you want? | 1 |
| 49 | Do you go on eating binges though you are not hungry? | 2 |
| 50 | On a scale of 0 to 5, where 0 means no restrained eating (eating whatever you want, whenever you want it) and 5 means total restraint (constantly limiting food intake and never 'giving in'), what number would you give yourself? | 1 |
| 51 | To what extent does this statement describe your eating behavior? 'I start dieting in the morning, but because of any number of things that happen during the day, by evening I have given up and eat what I want, promising myself to start dieting again tomorrow.' | 2 |

TFEQ, Three-Factor Eating Questionnaire

**Supplementary Table 2. Twenty-seven identified brain regions with large magnitude loading vectors.** The regions were sorted with their magnitudes. The information regarding the atlas index, name of regions, and behavioral domain was obtained from the Brainnetome atlas [43].

| **Atlas index** | **Region** | **Gyrus** | **Hemi-sphere** | **Canonical coefficient** | **Behavioral domain** |
| --- | --- | --- | --- | --- | --- |
| 137 | rostrodorsal area 39(Hip3) | Inferior Parietal Lobule | L | 0.4511 | Cognition |
| 135 | caudal area 39(PGp) | Inferior Parietal Lobule | L | 0.4198 | Cognition |
| 170 | ventral dysgranular and granular insula | Insular Gyrus | R | -0.3974 | Sensorimotor |
| 188 | subgenual area 32 | Cingulate Gyrus | R | -0.3337 | Reward |
| 48 | medial area 11 | Orbital Gyrus | R | 0.2927 | Reward |
| 106 | medioventral area37 | Fusiform Gyrus | R | -0.2056 | Visual |
| 58 | area 4(upper limb region) | Precentral Gyrus | R | 0.1893 | Sensorimotor |
| 127 | caudal area 7 | Superior Parietal Lobule | L | 0.1800 | Cognition |
| 147 | medial area 7(PEp) | Precuneus | L | 0.1651 | Default |
| 12 | medial area 9 | Superior Frontal Gyrus | R | -0.1402 | Reward |
| 61 | area 4(tongue and larynx region) | Precentral Gyrus | L | -0.1337 | Sensorimotor |
| 109 | rostral area 35/36 | Parahippocampal Gyrus | L | 0.1300 | Cognition |
| 88 | anterior superior temporal sulcus | Middle Temporal Gyrus | R | -0.1281 | Language |
| 205 | inferior occipital gyrus | lateral Occipital Cortex | L | -0.1268 | Visual |
| 230 | dorsolateral putamen | Basal Ganglia | R | -0.1142 | Motor |
| 189 | caudal lingual gyrus | MedioVentral Occipital Cortex | L | -0.0936 | Visual |
| 108 | lateroventral area37 | Fusiform Gyrus | R | -0.0671 | Language |
| 141 | caudal area 40(PFm) | Inferior Parietal Lobule | L | 0.0619 | Cognition |
| 87 | anterior superior temporal sulcus | Middle Temporal Gyrus | L | -0.0577 | Language |
| 211 | medial amygdala | Amygdala | L | -0.0483 | Emotion |
| 105 | medioventral area37 | Fusiform Gyrus | L | -0.0406 | Language |
| 155 | area 1/2/3(upper limb, head and face region) | Postcentral Gyrus | L | 0.0365 | Sensorimotor |
| 238 | rostral temporal thalamus | Thalamus | R | -0.0335 | Reward |
| 206 | inferior occipital gyrus | lateral Occipital Cortex | R | -0.0224 | Visual |
| 209 | lateral superior occipital gyrus | lateral Occipital Cortex | L | 0.0121 | Cognition |
| 80 | rostral area 22 | Superior Temporal Gyrus | R | -0.0077 | Language |
| 58 | area 4(upper limb region) | Precentral Gyrus | R | 0.1893 | Sensorimotor |

L, left; R, right.

**Supplementary Table 3. The coefficients of the loading vector for 19 identified questions in the TFEQ.** The questions were sorted based on their magnitudes. The explanations of each index are described in Supplementary Table 1.

| Index | Canonical coefficient | TFEQ factor |
| --- | --- | --- |
| 11 | 0.5149 | Disinhibition |
| 30 | 0.3482 | Dietary restraint |
| 48 | 0.3236 | Dietary restraint |
| 44 | 0.2879 | Dietary restraint |
| 50 | 0.2769 | Dietary restraint |
| 43 | 0.2585 | Dietary restraint |
| 33 | 0.2280 | Dietary restraint |
| 28 | 0.2263 | Dietary restraint |
| 32 | 0.2069 | Dietary restraint |
| 42 | 0.1673 | Dietary restraint |
| 6 | 0.1631 | Dietary restraint |
| 37 | 0.1592 | Dietary restraint |
| 18 | 0.1555 | Dietary restraint |
| 35 | 0.1272 | Dietary restraint |
| 9 | 0.0999 | Disinhibition |
| 27 | 0.0596 | Disinhibition |
| 40 | 0.0370 | Dietary restraint |
| 2 | 0.0306 | Disinhibition |
| 20 | 0.0204 | Dietary restraint |

TFEQ, Three-Factor Eating Questionnaire

**Supplementary Table 4. The top 27 frequently selected brain regions from 1000 bootstrapping.** The regions were sorted based on the frequency of selection. The regions that were common with those in the main results were highlighted as bold italic font.

| **Atlas index** | **Region** | **Gyrus** | **Hemi-sphere** | **Selection frequency** | **Behavioral domain** |
| --- | --- | --- | --- | --- | --- |
| ***188*** | subgenual area 32 | Cingulate Gyrus | R | 43.4% | Reward |
| ***170*** | ventral dysgranular and granular insula | Insular Gyrus | R | 42.3% | Sensorimotor |
| ***137*** | rostrodorsal area 39(Hip3) | Inferior Parietal Lobule | L | 40.9% | Cognition |
| ***135*** | caudal area 39(PGp) | Inferior Parietal Lobule | L | 40.8% | Cognition |
| ***106*** | medioventral area37 | Fusiform Gyrus | R | 40.0% | Visual |
| ***48*** | medial area 11 | Orbital Gyrus | R | 38.5% | Reward |
| ***127*** | caudal area 7 | Superior Parietal Lobule | L | 37.1% | Cognition |
| ***88*** | anterior superior temporal sulcus | Middle Temporal Gyrus | R | 32.3% | Language |
| ***147*** | medial area 7(PEp) | Precuneus | L | 31.0% | Default |
| ***105*** | medioventral area37 | Fusiform Gyrus | L | 30.7% | Language |
| ***155*** | area 1/2/3(upper limb, head and face region) | Postcentral Gyrus | L | 29.0% | Sensorimotor |
| ***205*** | inferior occipital gyrus | lateral Occipital Cortex | L | 27.1% | Visual |
| ***61*** | area 4(tongue and larynx region) | Precentral Gyrus | L | 26.9% | Sensorimotor |
| ***206*** | inferior occipital gyrus | lateral Occipital Cortex | R | 26.9% | Visual |
| 166 | ventral agranular insula | Insular Gyrus | R | 26.1% | Reward |
| ***230*** | dorsolateral putamen | Basal Ganglia | R | 26.1% | Motor |
| ***222*** | globus pallidus | Basal Ganglia | R | 25.4% | Sensorimotor |
| ***109*** | rostral area 35/36 | Parahippocampal Gyrus | L | 25.2% | Cognition |
| ***108*** | lateroventral area37 | Fusiform Gyrus | R | 24.9% | Language |
| 33 | caudal area 45 | Inferior Frontal Gyrus | L | 24.1% | Language |
| ***58*** | area 4(upper limb region) | Precentral Gyrus | R | 23.4% | Sensorimotor |
| 59 | area 4(trunk region) | Precentral Gyrus | L | 23.3% | Sensorimotor |
| ***87*** | anterior superior temporal sulcus | Middle Temporal Gyrus | L | 22.8% | Language |
| ***12*** | medial area 9 | Superior Frontal Gyrus | R | 22.7% | Reward |
| 143 | rostroventral area 39(PGa) | Inferior Parietal Lobule | L | 22.5% | Cognition |
| 159 | area 2 | Postcentral Gyrus | L | 22.5% | Sensorimotor |
| 127 | caudal area 7 | Superior Parietal Lobule | L | 37.1% | Cognition |

L, left; R, right.

**Supplementary Table 5. The top 19 frequently selected questions from 1000 bootstrapping.** The questions were sorted based on the frequency of selection. The questions that were common with those in the main results were highlighted as bold italic font. The index numbers are the same as those in Supplementary Table 1.

| Index | Selection frequency | TFEQ factor |
| --- | --- | --- |
| *11* | 77.0% | Disinhibition |
| *37* | 68.3% | Dietary restraint |
| *18* | 67.4% | Dietary restraint |
| *9* | 64.8% | Disinhibition |
| *50* | 64.6% | Dietary restraint |
| *48* | 57.8% | Dietary restraint |
| *6* | 56.9% | Dietary restraint |
| *32* | 56.6% | Dietary restraint |
| *27* | 55.8% | Disinhibition |
| *44* | 55.6% | Dietary restraint |
| *20* | 55.3% | Disinhibition |
| *33* | 55.2% | Dietary restraint |
| *28* | 54.2% | Dietary restraint |
| *43* | 53.6% | Dietary restraint |
| *40* | 52.9% | Dietary restraint |
| *30* | 52.7% | Dietary restraint |
| *42* | 47.7% | Dietary restraint |
| *35* | 42.5% | Dietary restraint |
| 49 | 40.5% | Disinhibition |

TFEQ, Three-Factor Eating Questionnaire

**Supplementary Table 6. Sixteen identified brain regions with large magnitude loading vectors in validation set.** The regions were sorted with their magnitudes. The information regarding the atlas index, name of regions, and behavioral domain was obtained from the Brainnetome atlas [43].

| **Atlas index** | **Region** | **Gyrus** | **Hemi-sphere** | **Canonical coefficient** | **Behavioral domain** |
| --- | --- | --- | --- | --- | --- |
| **5** | lateral area 9 | Superior Frontal Gyrus | L | 0.4209 | Cognition |
| **102** | caudoventral of area 20 | Inferior Temporal Gyrus | R | 0.4125 | Cognition |
| **101** | caudoventral of area 20 | Inferior Temporal Gyrus | L | 0.4065 | Cognition |
| **214** | lateral amygdala | Amygdala | R | 0.3937 | Reward |
| **32** | inferior frontal sulcus | Inferior Frontal Gyrus | R | 0.2745 | Cognition |
| **171** | dorsal granular insula | Insular Gyrus | L | 0.2734 | Reward |
| **154** | area 31 (Lc1) | Precuneus | R | -0.2537 | Cognition |
| **95** | intermediate lateral area 20 | Inferior Temporal Gyrus | L | 0.2236 | Cognition |
| **52** | lateral area 12/47 | Orbital Gyrus | R | 0.2043 | Reward |
| **193** | caudal cuneus gyrus | MedioVentral Occipital Cortex | L | 0.1075 | Cognition |
| **36** | rostral area 45 | Inferior Frontal Gyrus | R | 0.0809 | Cognition |
| **64** | caudal ventrolateral area 6 | Precentral Gyrus | R | 0.0658 | Sensorimotor |
| **157** | area 1/2/3(upper limb, head and face region) | Postcentral Gyrus | L | -0.0457 | Sensorimotor |
| **155** | area 1/2/3(upper limb | Postcentral Gyrus | L | -0.0374 | Sensorimotor |
| **63** | caudal ventrolateral area 6 | Precentral Gyrus | L | 0.0195 | Language |
| **36** | rostral area 45 | Inferior Frontal Gyrus | R | 0.0809 | Cognition |

L, left; R, right.

**Supplementary Table 7. The coefficients of the loading vector for eight identified questions in the EAT-26 in the validation set.** The questions were sorted based on their magnitudes.

| Index | Canonical coefficient | Question | EAT-26 factor |
| --- | --- | --- | --- |
| 25 | 0.6109 | I have the impulse to vomit after meals. | Diet |
| 10 | 0.4884 | I feel extremely guilty after eating. | Diet |
| 13 | 0.3811 | Other people think that I am too thin. | Oral control |
| 21 | 0.3531 | I give too much time and thought to food. | Bulimia & Food preoccupation |
| 9 | 0.3007 | I vomit after I have eaten. | Bulimia & Food preoccupation |
| 17 | 0.1336 | I eat diet foods. | Diet |
| 11 | 0.0992 | I am occupied with a desire to be thinner. | Diet |
| 18 | 0.0142 | I feel that food controls my life. | Bulimia & Food preoccupation |

EAT-26, Eating Attitudes Test
